# A Method Enabling High-Throughput Sequencing of Human Cytomegalovirus Complete Genomes from Clinical Isolates

**DOI:** 10.1371/journal.pone.0095501

**Published:** 2014-04-22

**Authors:** Steven Sijmons, Kim Thys, Michaël Corthout, Ellen Van Damme, Marnix Van Loock, Stefanie Bollen, Sylvie Baguet, Jeroen Aerssens, Marc Van Ranst, Piet Maes

**Affiliations:** 1 Laboratory of Clinical Virology, Rega Institute for Medical Research, Katholieke Universiteit Leuven, Leuven, Belgium; 2 Janssen Infectious Diseases BVBA, Beerse, Belgium; Temple University School of Medicine, United States of America

## Abstract

Human cytomegalovirus (HCMV) is a ubiquitous virus that can cause serious sequelae in immunocompromised patients and in the developing fetus. The coding capacity of the 235 kbp genome is still incompletely understood, and there is a pressing need to characterize genomic contents in clinical isolates. In this study, a procedure for the high-throughput generation of full genome consensus sequences from clinical HCMV isolates is presented. This method relies on low number passaging of clinical isolates on human fibroblasts, followed by digestion of cellular DNA and purification of viral DNA. After multiple displacement amplification, highly pure viral DNA is generated. These extracts are suitable for high-throughput next-generation sequencing and assembly of consensus sequences. Throughout a series of validation experiments, we showed that the workflow reproducibly generated consensus sequences representative for the virus population present in the original clinical material. Additionally, the performance of 454 GS FLX and/or Illumina Genome Analyzer datasets in consensus sequence deduction was evaluated. Based on assembly performance data, the Illumina Genome Analyzer was the platform of choice in the presented workflow. Analysis of the consensus sequences derived in this study confirmed the presence of gene-disrupting mutations in clinical HCMV isolates independent from *in vitro* passaging. These mutations were identified in genes RL5A, UL1, UL9, UL111A and UL150. In conclusion, the presented workflow provides opportunities for high-throughput characterization of complete HCMV genomes that could deliver new insights into HCMV coding capacity and genetic determinants of viral tropism and pathogenicity.

## Introduction

Human cytomegalovirus (HCMV), the prototype member of the herpesvirus subfamily *Betaherpesvirinae*, is a ubiquitous virus with seroprevalences ranging from 45 to 100% in the adult population [1]. Primary infection or reactivation usually remains asymptomatic; however, the virus can cause serious illness in newborns and immunosuppressed individuals such as transplant recipients and AIDS patients [2]. HCMV has the largest genome of all human herpesviruses, with a size of approximately 235 kbp. The genome consists of two unique fragments, the unique long (UL) and unique short (US) regions, which are both flanked by a pair of inverted repeats, termed terminal/internal repeat long (TRL/IRL) and internal/terminal repeat short (IRS/TRS). Four genomic isomers are present in equimolar concentrations through inversion of UL and US relative to each other [3].

The first complete genome sequence of HCMV, derived from the highly passaged laboratory strain AD169, was published in 1990 with 208 open reading frames (ORFs) predicted as protein-encoding [4]. Through comparison of different laboratory strains and isolates passaged more moderately on cultured human fibroblasts, it has been well established that AD169 contains major genome rearrangements. These affect a region at the 3′ end of the UL region, commonly referred to as the UL/b’ region, resulting in the loss of a 15 kbp fragment which encodes 19 additional ORFs [5], [6]. The HCMV genetic map was further refined by genome comparisons with chimpanzee cytomegalovirus and full genome sequencing of a handful additional clinical isolates [7]–[10]. The current HCMV genetic map as annotated on the HCMV reference sequence Merlin (NC_006273 [10]) contains 170 genes, some of which are only defined theoretically. In fact, recent publications defining the HCMV transcriptome have drawn a very sophisticated picture including alternative splicing and antisense transcription, which could redefine our understanding of the HCMV coding capacity [11]–[13]. The functionality of these products still awaits further confirmation. The determination of the complete genome sequence of additional, clinically representative isolates could assist in a better definition of the HCMV genetic map through comparative genomic approaches.

During the last years, next-generation sequencing (NGS) has immensely impacted the genomics field [14]. Although several complete HCMV genomes have been determined using the traditional cloning and Sanger sequencing approaches, it is still highly laborious and not suitable for high-throughput applications [4], [9], [10], [15]. NGS technology obviates the need for cloning procedures by the generation of enormous amounts of short sequence reads starting from minimal input material. The benefits of NGS for HCMV genomics were first demonstrated through the elucidation of variants present in laboratory preparations of the AD169 and Towne strains [16]. In an attempt to evaluate the effectiveness of NGS with clinical HCMV isolates, Cunningham *et al.* compared a more traditional PCR-based amplification and Sanger sequencing approach with a NGS approach using the Illumina Genome Analyzer (IGA; Illumina, Inc., San Diego, USA) [17]. In addition, the 454 GS FLX (Roche Applied Science, Penzberg, Germany) platform was successfully used to determine the first complete genome sequence of an Asian HCMV isolate [18]. Cunningham *et al.* showed that sequencing of complete HCMV genomes directly from clinical material is achievable, but given the small fraction of viral DNA, not practically amenable to high-throughput. In order to achieve a high-throughput application with NGS technology, a protocol to amplify and isolate highly pure viral DNA is desirable.

Currently, 33 complete HCMV sequences are available in the NCBI GenBank (v196.0), including 17 derived from unpassaged or moderately passaged material (up to 10 cell culture passages). Additional sequences of clinical isolates are necessary to better apprehend the genetic diversity and coding capacity of HCMV strains. Since sequencing complete genomes of clinically representative HCMV isolates in high-throughput awaits new amplification protocols, we have developed a dedicated amplification, sequencing and analysis workflow for HCMV genome characterization. The workflow maximizes sequencing capacity through the generation of highly pure HCMV DNA (>90% viral DNA). The efficiency of using 454 GS FLX and/or IGA for HCMV full genome sequencing was compared. Using a series of validation experiments, we show that consensus sequences derived by the workflow are representative for the strain present in the original clinical isolate. The presented workflow enables high-throughput analysis of HCMV full genome sequences and could serve as an important tool in elucidating the genetic diversity of this complex herpesvirus.

## Materials and Methods

### Patient Samples, Viruses and Cell Culture

Seven PCR-confirmed HCMV-positive urine samples were included in the study (primers listed in Table S1). Sample BE/9/2010 was taken from a child with a primary infection presenting with fever. Samples BE/10/2010 i1 and BE/10/2010 i2 were collected on the same day from a congenitally infected infant that was asymptomatic at birth. Sample BE/11/2010 was obtained from a child with a primary infection with liver dysfunction. Sample BE/21/2010 was taken from a pulmonary transplant recipient who had received a transplant and seroconverted in 2007. Finally, samples BE/27/2010 i1 and BE/27/2010 i2 were collected from a patient receiving a renal transplant in 2008 and seroconverting in 2009.

Typically, 1 mL of urine was centrifuged for 10 min at 300×g and the supernatant was subsequently filtered through a 0.45 µm filter (Minisart NY25, Sartorius AG, Göttingen, Germany). A confluent monolayer of human embryonic skin-muscle fibroblast cells (E_1_SM [19]) in a 25 cm^2^ flask containing 10 mL of DMEM (Life Technologies, Carlsbad, USA) supplemented with 10% fetal bovine serum (FBS, Life Technologies) was inoculated with 0.5 mL of the filtrate and incubated at 37°C in a humidified 5% CO_2_ environment. Infected cells were passaged every two weeks by diluting cells 1∶2 into a 75 cm^2^ flask after trypsination (0.05% Trypsin-EDTA, Life Technologies).

Strain Merlin was obtained from ATCC (ATCC-VR-1590, Lot Nr. 58730771, passage 4). A confluent monolayer of E_1_SM cells in a 75 cm^2^ flask containing 10 mL of DMEM was inoculated with 0.5 mL of the virus stock and the cells were incubated at 37°C and 5% CO_2_. After 1 h, the medium was removed and the cells were washed with 1X PBS (Life Technologies) before adding DMEM with 10% FBS.

### Viral DNA Purification and Multiple Displacement Amplification

Since clinical isolates do not produce large amounts of cell-free virus, a procedure was needed to purify intracellular, viral DNA from large backgrounds of cellular DNA. We therefore adapted a protocol described by Sinzger *et al.* [20]. Briefly, cells from three 75 cm^2^ flasks were trypsinized and pooled. After lysis in a Tris buffer containing sucrose and Triton X-100, cellular DNA was digested using micrococcal nuclease (Thermo Fisher Scientific, Waltham, USA). Subsequently, DNA was extracted using the QIAamp DNA Blood Mini Kit. Extracted DNA was amplified by multiple displacement amplification using the REPLI-g Mini Kit (Qiagen, Hilden, Germany). For each sample, three independent REPLI-g reactions were pooled. A mixture of 150 µL of REPLI-g products, 300 µL of pure ethanol and 15 µL of 3 M sodium acetate was incubated at −80°C for 2 h. The samples were centrifuged for 30 min at 20,000×g (4°C), the supernatant was removed and the pellets were washed with 70% ethanol. Afterwards, the samples were centrifuged again for 30 min at 20,000×g (4°C) and the supernatant was removed. The pellets were air dried and resuspended in 50 µL of QIAamp Elution Buffer (Qiagen).

For the purification of an unpassaged isolate, 200 mL of sample BE/21/2010 was centrifuged for 10 min at 300×g and the supernatant was centrifuged for 2 h at 100,000×g (4°C) in a type 35 rotor (Beckman Coulter Inc., Brea, USA). The pellet was resuspended in 200 µL of 1X PBS and DNA was extracted using the QIAamp DNA Blood Mini Kit (Qiagen). Extracted DNA was amplified through whole genome amplification as described above.

### Quantitation of Viral and Cellular DNA

Viral and cellular DNA contents were evaluated using a quantitative PCR assay (qPCR). HCMV DNA was quantitated through amplification of a fragment of the conserved major capsid protein-encoding gene UL86. For human DNA, a region of the β-globin household gene was amplified. Primers and probes were obtained from Eurogentec (Liège, Belgium); the sequences are listed in Table S1. The qPCR was carried out using TaqMan Universal PCR Master Mix (Life Technologies) on an Applied Biosystems 7500 Fast Real-Time PCR system (Life Technologies), following the manufacturer’s protocols. Both standards and samples were quantitated in duplicate, viral and cellular DNA was quantitated in separate wells.

For absolute quantitation, standard series were produced by serial dilution of HCMV UL86 and human β-globin standards. The standards were prepared through PCR amplification of the qPCR targets and products were gel purified using the QIAquick Gel Extraction Kit (Qiagen). After spectrophotometrical quantitation with a BioPhotometer (Eppendorf, Hamburg, Germany), DNA concentrations were converted to copy number/µL using the formula described by Fronhoffs *et al.* [21]. Viral and cellular DNA copy numbers were converted to absolute weight (µg of DNA) for mutual comparison.

### Next-generation Sequencing

For 454 GS FLX sequencing, total DNA was fragmented to an average length of 400 bp using a Covaris E210 system (Covaris). DNA fragments were end-repaired, 3′-adenylated, ligated to adapters (GS FLX Titanium Rapid Library MID Adaptors kit, Roche) and size-selected (>350 bp) using the SPRIworks Fragment Library System II (Beckman Coulter Genomics). The quality of the library was evaluated using a high-sensitivity DNA chip on a model 2100 Bioanalyzer (Agilent Technologies). Libraries were quantitated with the TBS-380 Mini-Fluorometer (Promega) and subsequently pooled at equimolar concentrations. Prior to sequencing, clonal amplification was performed during an emulsion based PCR (GS FLX Titanium emPCR Kit, Roche). Sequencing was performed using the GS FLX Titanium Sequencing Kit (Roche). Following sequencing, processing of the raw sequence data was performed with the Roche Sequencing System Software package.

For Illumina Genome Analyzer (IGA) sequencing, total DNA was fragmented to an average length of 200 bp using a Covaris E210 system (Covaris). The ends of the fragmented DNA were repaired, adenylated and Illumina compatible adaptors (Index PE Adaptor Oligo Mix, Illumina) were ligated using the SPRIworks Fragment Library System I (Beckman Coulter Genomics). Fragments were indexed using the Multiplexing Sample Preparation Oligonucleotide Kit (Illumina) and the library was enriched during 12 PCR cycles. Enriched fragments were visualized on a Bioanalyzer (Agilent Technologies) for quality control and quantitation. Finally, samples were pooled at equimolar ratios and put on the Illumina cluster station for cluster generation using the TruSeq PE Cluster Kit v2 (Illumina). One hundred and nine cycles of multiplexed paired-end sequencing were performed using the TruSeq SBS Kit v5 (Illumina). Following sequencing on the GAIIx, processing of the raw sequence data was performed with the Illumina analysis software (Casava 1.7.0).

### Assembly of Consensus Sequences and Finishing with Sanger Sequencing

IGA and 454 GS FLX datasets were first subjected to a quality control step using *QUASR* v6.08 (http://sourceforge.net/projects/quasr/). Low-quality bases were trimmed from the 3′ end of reads until the median quality of the reads was higher than 20. Reads smaller than 20 bp were removed. A *de novo* assembly was constructed for 454 GS FLX reads using *MIRA* v3.4.1.1 [22], [23] with assembly settings on ‘accurate’ and for IGA reads with *Velvet* v1.2.07 [24]. The hash value, expected coverage and coverage cutoff parameters needed for Velvet assemblies were first estimated using VelvetOptimiser (Perl script, http://bioinformatics.net.au/software.velvetoptimiser.shtml) and then manually adjusted to produce longer contigs (Table S2). The resulting contigs were assembled using *Phrap* v1.090518 [25] and *Phrap* contigs longer than 1,000 bp were included in a NCBI nucleotide *BLAST* search to find a suitable HCMV reference sequence. Next, all *Phrap* contigs were aligned to the selected reference sequence using *NUCmer* included in the *MUMmer 3* package [26]. For this alignment, reference sequences were trimmed of its terminal repeats, except for the 50 bp region adjacent to UL and US regions, as described [17]. This alignment was used to build a hybrid reference combining *Phrap* contigs and pieces of the original reference sequence in regions where no contigs had mapped. Finally, a reference assembly was constructed using the 454 GS FLX and IGA reads with *MIRA* and a consensus sequence of the strain was generated. This assembly was outputted in ACE format and visualized using *Tablet* v1.12.12.05 [27], [28]. The complete consensus sequence was manually inspected and any misassembly corrected.

Gaps remaining in the consensus sequences after assembly of NGS data were resolved through PCR amplification and Sanger sequencing. PCR reactions were carried out with HotStarTaq DNA Polymerase (Qiagen) using standard manufacturer’s protocols. Primer sequences and annealing temperatures are presented in Table S3. Products were cleaned up before sequencing with ExoSAP-IT (Affymetrix, Santa Clara, USA). Sequencing reactions were performed in both directions using the BigDye Terminator v3.1 Cycle Sequencing Kit (Life Technologies) and sequencing products were analyzed in an ABI PRISM 3100 Genetic Analyzer. Chromatograms were interpreted and contigs were joined with the complete genome consensus using *Lasergene SeqMan* v7.0.0 (DNASTAR, Madison, USA).

Using the final complete genome consensus sequence, a reference assembly was constructed using the *CLC Genomics Workbench* v5.5.1 (CLC bio, Aarhus, Denmark) and the regions corresponding to gaps in the original reference mapping were revised. The final consensus sequences were submitted to NCBI GenBank (accession no. KC519319–KC519323). Sequence alignments were constructed using *MAFFT* v6.903 [29] and visualized with *MEGA5* [30]. Comparisons with other HCMV strains included NCBI GenBank entries GU179001, BK00039, FJ527563, AC146999, AY315197, AC146851, FJ616285, GU937742, AC146905, AC146907, AC146904, AC146906, EF999921, GQ221974, GQ466044, GU179291, GQ221973, GQ396663, GQ396662, GQ221975, GU179288, GU179290, GU179289, HQ380895, JN379814, JN379815 and JN379816.

To assess the content of the original sample preparations, a *de novo* assembly was built with the *CLC Genomics Workbench* using only 454 GS FLX and IGA reads that were not mapped onto the final HCMV reference sequence. The resulting contigs were analyzed with the blastn command of the *BLAST+* application [31] using the complete nucleotide (nt) database. Output of contig database searches was interpreted with *MEGAN4* [32].

### Evaluation of Sequencing Technologies and Assembly Software

To evaluate the performance of assemblies using only 454 GS FLX, IGA or both read sets, *de novo* assemblies were constructed and the resulting contigs were aligned with *NUCmer* against the final genome sequence of the appropriate strain to evaluate genome coverage. The freeware software suites (*MIRA*, *Velvet* and *Phrap*) that were used to build the initial consensus sequences were compared to a commercial alternative (*CLC Genomics Workbench*). Statistical analyses were performed with *SPSS Statistics* v21 (IBM, Armonk, USA). Overall differences of n50 contig length and number of gaps left in the assembly were tested using the Friedman Test and individual groups were compared using the Wilcoxon Signed Ranks Test with Bonferroni correction.

## Results and Discussion

### Development of a Sample Preparation Protocol Generating Highly Pure HCMV DNA

**Figure 1:**
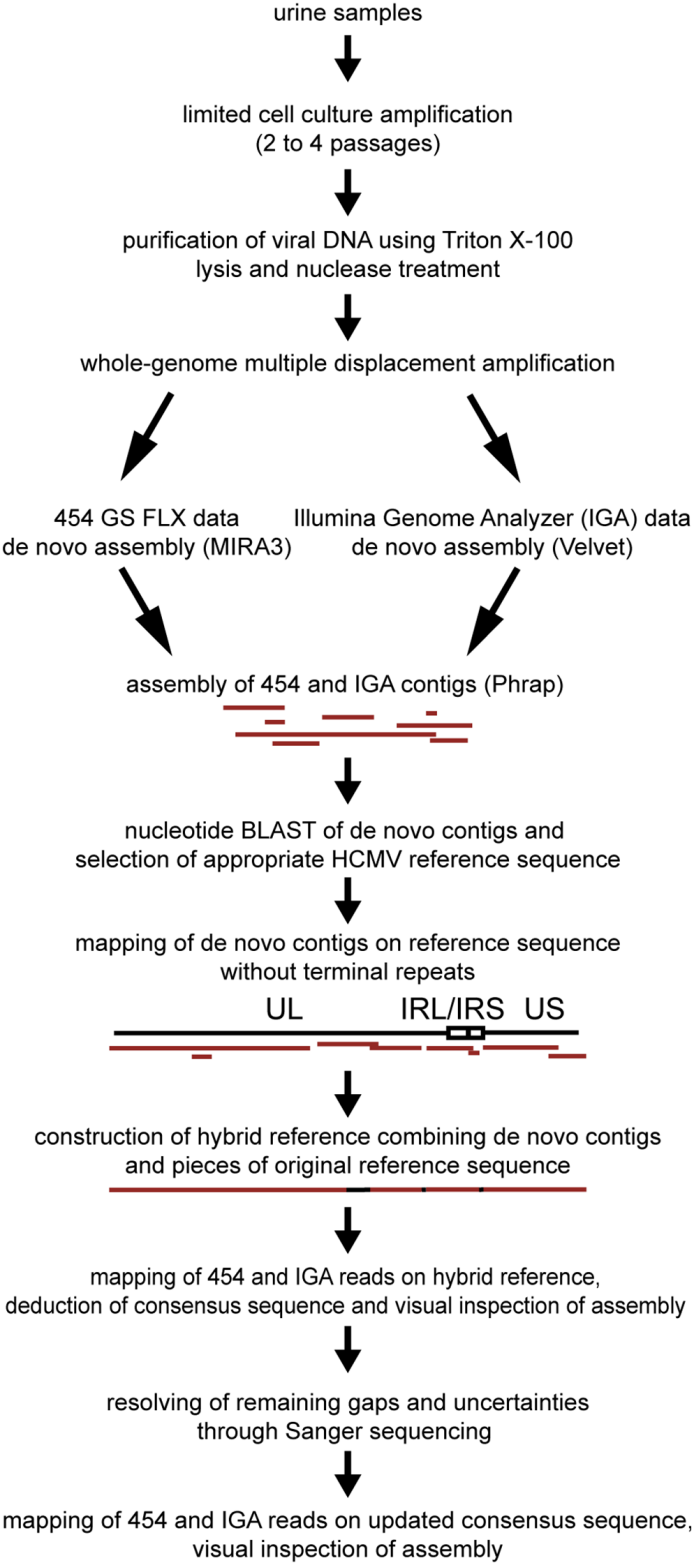
Schematic overview of the amplification, sequencing and analysis workflow. UL and US denote unique and unique short regions of the genome; IRL and IRS denote internal repeats.

**Figure 2:**
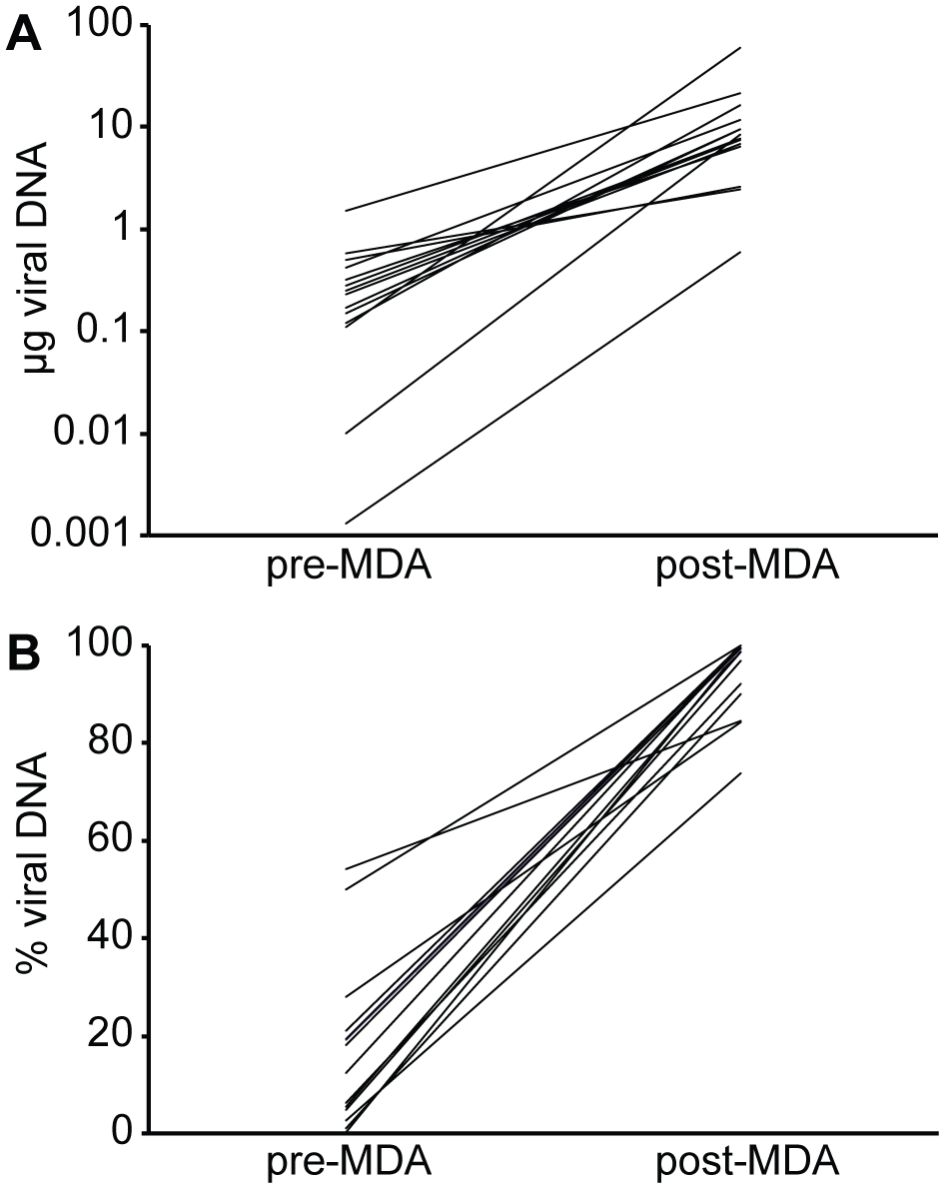
Multiple Displacement Amplification (MDA) selectively amplifies viral but not cellular DNA. Amounts of viral and cellular DNA were estimated using qPCR before and after amplification of the DNA extraction products using MDA (pre- and post-MDA). In [A], the increase in absolute amounts of viral DNA (µg) is visualized, [B] represents the relative increase of viral to cellular DNA (% viral DNA).

For the development of a method to characterize HCMV genomes in high-throughput, a procedure was needed to amplify the viral material in clinical samples. To maximize sequencing capacity, extract purity had to be optimized. PCR-based amplification approaches that use a set of conserved primers covering the complete HCMV genome have been applied [17], [33]. However, the labor-intensity of these methods compromises a high-throughput perspective. Therefore, we have amplified viral material through passaging isolates on E_1_SM cells, a human fibroblast cell line (Figure 1). The number of passages on fibroblasts in this amplification was limited to avoid potential genetic adaptation of HCMV to growth on fibroblasts [34]. This implied that virus production would be low and predominantly cell-associated. Amplification by passaging HCMV clinical isolates on fibroblasts had already been used as a preparative step for NGS analysis, but usually DNA was isolated through a whole cell extraction [17], [18]. The extracted viral DNA is usually heavily contaminated with cellular DNA, which impacts on the efficiency of the sequencing process. We chose to implement a technique to specifically purify cell-associated viral DNA [20]. This method is based on lysis of the cellular membranes and nuclease-based cleavage of cellular DNA, followed by extraction of viral DNA from nucleocapsids. Isolates were harvested at passage 2 or 4, when the first foci of cytopathogenic effect became visible. To assess viral yield and purity, we developed a quantitative PCR to evaluate the amounts of viral and cellular DNA present in the isolates. After virus isolation and DNA extraction, viral DNA yield and especially purity were considered unsatisfactory for NGS, since most samples (11/14) contained less than 500 ng of HCMV DNA and the majority of the DNA detected was of cellular origin (Figure 2, pre-MDA). To further amplify viral DNA, samples were subjected to multiple displacement amplification (MDA). MDA makes use of the high processivity, strand displacement and proofreading capacity of the Φ29 DNA polymerase to amplify DNA using random primers. This method can amplify nanograms of DNA to micrograms and generates long contiguous strands with very low mutation rates (10^−6^). Amplification biases have been reported, but tend to be more problematic when starting from very low amounts of input material such as in single-cell sequencing [35]. MDA affected both yield and purity of viral DNA, with amounts of viral DNA mostly above 5 µg (11/14) and purities largely higher than 90% (11/14) (Figure 2, post-MDA). Only for the unpassaged isolate of strain BE/21/2010, viral DNA yield remained low with 600 ng of HCMV DNA, but with an estimated purity of 85%. The relative increase in viral DNA contents after MDA could possibly be attributed to the differential quality of viral and cellular DNA after nuclease treatment. While viral DNA was protected from nuclease activity by the viral capsid, cellular DNA was presumably heavily degraded. Although cellular DNA is still detected by the qPCR assay, which only amplifies a 167 bp fragment, we hypothesize that it is amplified much less efficiently by MDA than the intact viral DNA [36]. Because of the observed increase in viral DNA yield and purity, genomic contents of extracts could be characterized more efficiently, supporting high-throughput applications.

### Sequencing and Assembly of HCMV Genomes Using 454 GS FLX and/or IGA Data

Purified HCMV DNA was analyzed using both 454 GS FLX and IGA to compare the performance of both systems in generating consensus sequences of complete genomes. Although several complete genome sequences of HCMV strains are available on NCBI GenBank, substantial regions of the genome are highly variable, which makes a mapping assembly unsuitable for analysis of distinct HCMV strains. Mapping assemblies left large areas of the genome uncovered. A large fraction of the unmapped reads, however, were found to be genuine HCMV reads with *BLAST* (data not shown). Therefore, a *de novo* assembly approach was chosen, followed by scaffolding of *de novo* contigs on HCMV reference sequences (Figure 1). A similar assembly approach using different software suites was already successfully implemented for HCMV [17]. Briefly, *de novo* contigs were mapped on HCMV reference genomes and a hybrid reference sequence was constructed combining contigs and pieces of the original reference sequence in regions with no contig coverage. Subsequently, the consensus sequence was derived from a mapping assembly of all sequencing reads against this hybrid reference.

The final consensus sequences were used to construct a reference assembly using 454 GS FLX and IGA datasets. The percentage of reads mapped to the HCMV consensus sequence was generally in accordance with the sample purity predicted by the qPCR assay (Table 1). Since qPCR assays only quantified cellular DNA as a possible contaminant, this measure could overestimate sample purity, but there was only a small difference between qPCR and read mapping purity estimates for most samples (9/14<5%, 11/14<10%). Only strains BE/21/2010 UP and BE/27/2010-1 showed a large discrepancy (>20%) between the purity estimates, with the actual amount of reads that mapped to the HCMV consensus much lower than expected by qPCR. This discrepancy could be explained by the fact that qPCR assays only detect one segment of viral and cellular DNA, while the sequencing data reflect total DNA levels. To identify additional contaminating DNA present in the isolates, *de novo* assemblies were performed using 454 GS FLX and IGA reads that did not map to the HCMV consensus sequence. These contigs were analyzed using *BLAST* (Table S4). For strain BE/27/2010-1, only the presence of human DNA could account for the discrepancy between qPCR and read mapping results. The unmapped reads of BE/21/2010 UP largely consisted of human DNA and some bacterial and papillomaviral sequences. With only 12% of NGS reads being HCMV-specific for BE/21/2010 UP, we essentially encountered the same limitations as Cunningham and colleagues [17] for sequencing of unamplified clinical material and confirm that this is currently not amenable to high-throughput applications, even after MDA. This result indicates that an amplification and/or enrichment procedure for viral DNA is crucial to efficiently utilize NGS high-throughput capacities, which is provided through our cell culture extraction and MDA workflow. For three other samples, a small number of HCMV sequences were detected that did not map to the consensus sequence during the reference assembly (Table S4). Nevertheless, these contigs, mostly smaller than 1,000 bp, could be aligned to the consensus using the *NUCmer* algorithm with similarities close or equal to 100% (data not shown).

**Table 1:** Mapping of 454 GS FLX and IGA reads to strain consensus sequences.

Strain	GenBank accession	Isolate and/or passage number	# reads mapped	# reads unmapped	% reads mapped	qPCR sample purity	Average read depth (454 GS FLX + IGA)
Merlin	NC_006273		5,855,670	76,782	99	100	1306 (23+1283)
BE/9/2010	KC519319	p2	7,166,157	351,662	95	100	1611 (43+1568)
		p5	8,934,863	226,933	98	100	1978 (19+1959)
		p7	8,445,946	607,953	93	99	1879 (28+1851)
		p11	6,781,195	1,542,530	81	74	1507 (22+1485)
BE/10/2010	KC519320	i1 p2	10,359,782	63,203	99	100	2262 (22+2240)
		i2 p2	5,963,342	50,527	99	100	1314 (27+1287)
BE/11/2010	KC519321	p2	8,855,022	325,142	96	99	1971 (30+1941)
		p5	9,205,907	470,107	95	100	2046 (26+2020)
		p9	5,751,100	682,788	89	92	1275 (13+1262)
BE/21/2010	KC519322	up	5,429,700	39,097,554	12	85	1077 (0+1077)
		p4	6,008,424	209,938	97	84	1390 (64+1326)
BE/27/2010	KC519323	i1 p4	1,190,000	2,150,782	36	90	273 (14+259)
		i2 p4	1,256,717	89,568	93	97	328 (44+284)

i = isolate number.

p = passage number.

up = unpassaged.

Since both immunocompetent and immunocompromised patients can be co-infected by and shed multiple HCMV strains, the derived consensus sequences do not necessarily represent a single, contiguous genome but a collection of the most abundant variants at each position in the genome [33], [37], [38]. However, inspection of our assemblies always showed the predominance of a single variant throughout the entire genome, without any clear evidence of multiple infections, suggesting that these particular consensus sequences do represent contiguous strain sequences (data not shown).

**Figure 3:**
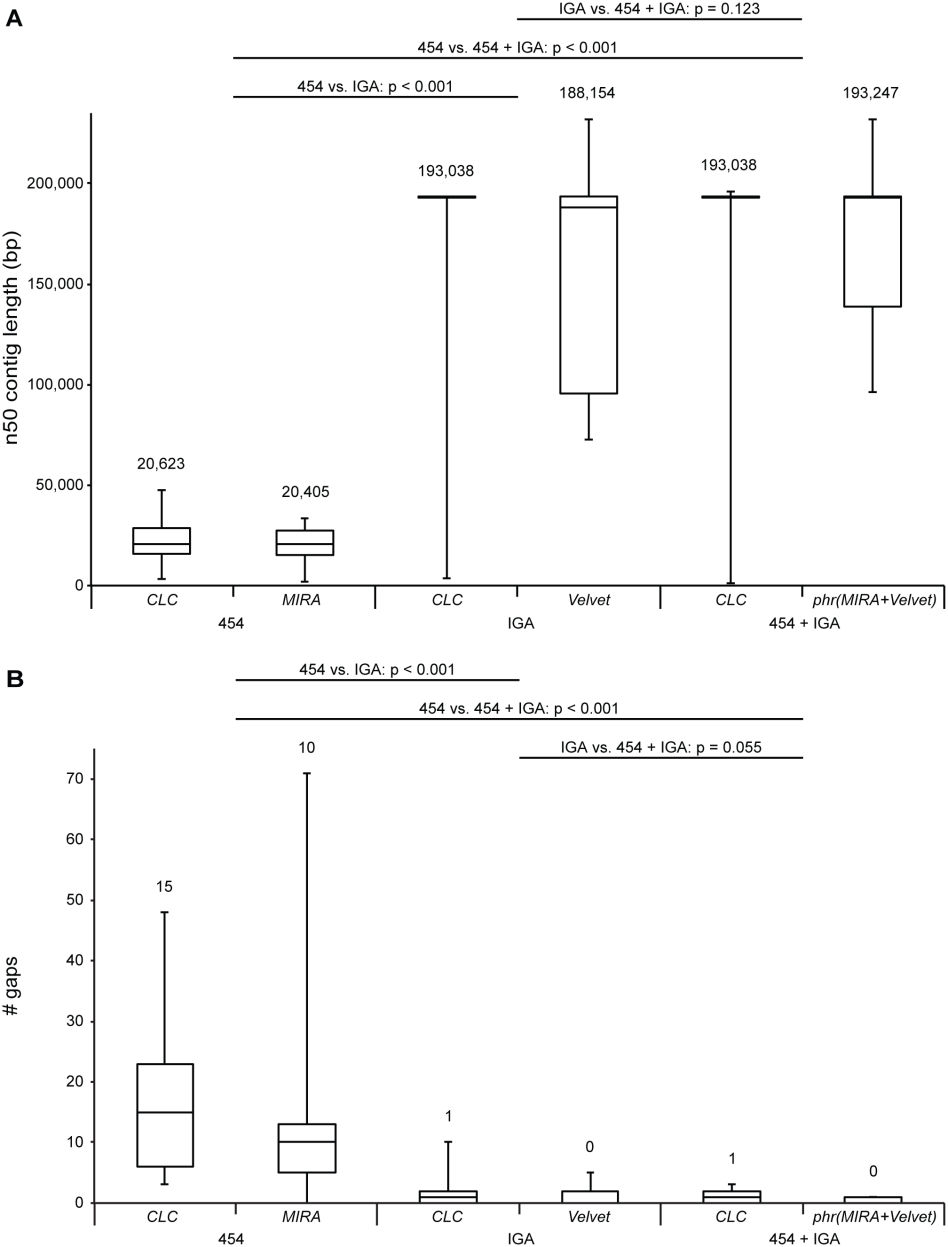
Assembly performance using 454 GS FLX, IGA or both and freeware or commercial software suites. Boxplots representing [A] the range of n50 contig lengths and [B] number of gaps in contig coverage of consensus sequences after *de novo* assembly of respectively 454 GS FLX, IGA or combined datasets. The central line in the box represents the median, top and bottom represent the 75 and 25 percentile and error bars represent minimum and maximum values. Median values are stated above each boxplot. Datasets (454 GS FLX and/or IGA) and software suites (*CLC Genomics Workbench*, *MIRA*, *Velvet* or *Phrap* combining *MIRA* and *Velvet* assemblies) are indicated below the plots. Since normality was violated, overall differences for n50 contig length and number of gaps were tested with the non-parametric Friedman test (n = 13; n50 contig length: χ^2^(5) = 42.506, p<0.001; gaps: χ^2^(5) = 37.275, p<0.001). Comparisons between assemblies based on different datasets were made using the Wilcoxon Signed Ranks Test with Bonferroni correction; p-values are reported in the figure. Because of the Bonferroni correction, differences are only significant when p<0.017.

To compare the utility of 454 GS FLX and IGA datasets in the characterization of HCMV genomes, *de novo* assemblies were constructed using only 454 GS FLX or IGA data and a combination of both. A commercial package, the *CLC Genomics Workbench*, was compared to *MIRA* and *Velvet*, which are freely available. *MIRA* was used for assembly of 454 GS FLX data, while IGA data were assembled with *Velvet*. *Velvet* uses a de Bruijn graph strategy which is better suited for large datasets than the overlap-layout-consensus strategy that *MIRA* utilizes [39]. Both datasets were combined through a *Phrap* assembly of combined contigs. The performance of *de novo* assemblies was compared by mapping the resulting contigs on the appropriate consensus sequence that was derived earlier. A complete overview of results for each dataset is presented in Table S2. Here, we present the range of n50 contig lengths (Figure 3a) and number of gaps left when contigs were mapped to the consensus sequence (Figure 3b). The n50 contig length states that 50% of the entire assembly is comprised in contigs equal to or larger than this length. These data clearly illustrate that IGA datasets produce assemblies that are comparable to those using both datasets combined. Both n50 contig length and number of gaps do not significantly differ between both cases (Wilcoxon Signed Ranks Test; n50 contig length: p = 0.123; gaps: p = 0.055). The n50 contig length is drastically lowered by using only 454 GS FLX data, which consequently increases the number of gaps left after the initial assembly (Wilcoxon Signed Ranks Test; n50 contig length: p<0.001; gaps: p<0.001). Our results show that IGA datasets outperform 454 GS FLX datasets. IGA sequencing has a higher throughput and lower cost per base and therefore achieves much higher coverages than 454 GS FLX sequencing in this study (Table 1). The benefits of this higher coverage clearly outweigh the longer length of 454 GS FLX reads for *de novo* HCMV genome assembly. In fact, the combined use of 454 GS FLX and IGA datasets does not significantly alter *de novo* contig length. Taking into account the higher error rates of 454 GS FLX sequencing in homopolymeric stretches [40], IGA would be the preferred platform of both for high-throughput sequencing of HCMV isolates.

Based on n50 contig lengths, commercially and freely available software packages delivered no significantly different assemblies (Wilcoxon Signed Ranks Test; p = 0.933). There was, however, a small but significant difference in the number of gaps left in the assembly, with the freeware assemblies containing less gaps (Wilcoxon Signed Ranks Test; p = 0.031). When IGA data were involved, the assemblies produced by the *CLC Genomics Workbench* showed a smaller range in n50 contig lengths than *Velvet* assemblies. Assembly of IGA data using the *CLC Genomics Workbench* is in fact more user-friendly than *Velvet* assemblies that have to be optimized manually by adjusting several parameters. This optimization step makes these assemblies less reproducible. Recently, novel freeware *de novo* assembly algorithms have been released that show improved performance and could be better alternatives to the commercial assembly options than *Velvet* [41]–[44].

### Consensus Sequences are Representative for the HCMV Population Present in the Original Clinical Isolate

Four different approaches were combined to validate the consensus sequences that were generated using our preparation, sequencing and assembly pipeline. (1) Reference strain Merlin was resequenced and (2) consensus sequences of independent isolates of the same patient (BE/10/2010 and BE/27/2010) were compared. (3) Strain BE/21/2010 was sequenced both directly from clinical material and after cell culture passage to evaluate how the consensus sequence was altered during cell culture adaptation. (4) Finally, strains BE/9/2010 and BE/11/2010 were sampled at different culture passages (2–11 passages) to characterize potential changes in the consensus sequence during further adaptation to cell culture.

(1) To validate our workflow, the HCMV reference strain Merlin was grown for one additional passage and harvested using the aforementioned protocol. The consensus sequence was generated using a *de novo* approach and the original reference sequence was only used to guide assembly of *de novo* contigs. The generated consensus sequence was aligned to the original reference [10]. Only two SNPs were detected between both sequences. The first SNP was situated in gene UL32, encoding the major tegument protein pp150, resulting in a silent CTC to CTG substitution at amino acid position 1,038. When the read alignment of the assembly was inspected, this mutation was observed in 65% of reads, with the other 35% still displaying the wild-type G. Another SNP, a G to C substitution, was initially noted in the IRL at nucleotide position 195,063. However, when variants that were segregated between IRL and TRL copies were added up, it was noted that only 24% of reads contained this substitution. Interestingly, these two substitutions were also noted when Merlin was cloned into a BAC and resequenced by Stanton and colleagues [45]. They reported the substitution in UL32 to be a single nucleotide polymorphism in the original Merlin population. The fact that these SNPs were also found using our workflow confirms that these were present in the original viral population.

(2) To assess the reproducibility of our consensus-generating pipeline, we independently passaged twice two samples taken from the same patient on the same day (BE/10/2010 i1 and BE/10/2010 i2) and subsequently purified, sequenced and assembled the genomes. After analysis, nearly identical consensus sequences were obtained with only a minor length difference in three homopolymer regions (Table S5). Likewise, strains BE/27/2010 i1 and BE/27/2010 i2 were derived from sequential isolates of the same patient, derived with an interval of 49 days. Both samples were independently passaged four times in E_1_SM cells and processed in our workflow after which consensus sequences were compared. Sequences only differed in the length of one homopolymer region (Table S5). All apprehensive homopolymer regions were situated in non-coding regions. These findings show that the generated consensus sequences are reproducible and furthermore indicate that the consensus sequence of strain BE/27/2010 remained stable during 49 days of intrahost viral replication.

(3) Strain BE/21/2010 was isolated and sequenced directly from HCMV-positive urine and simultaneously passaged four times in E_1_SM cells to characterize potential changes in the consensus sequence during initial adaptation to cell culture. A substitution was detected in gene UL30 (A13G) in 45% of reads derived from the passaged isolate. Differences between both consensus sequences were only situated in the length of four homopolymer regions and one trinucleotide repeat (Table 2). These regions also display variable lengths in different HCMV strains. Furthermore, a closer inspection of the assembly in these regions revealed some repeat length heterogeneity in NGS reads. This could both reflect technological constraints in the prediction of homopolymer lengths and intrapatient variability in repeat lengths. Given the fact that these repeats are mostly situated in non-coding sequences and these regions are inherently of variable length in different isolates, it seems perfectly conceivable that intrapatient heterogeneity exists. In fact, the length difference in the trinucleotide repeat cannot be explained by homopolymer error and thus probably reflects intrapatient heterogeneity.

**Table 2:** Comparison of strain BE/21/2010 consensus sequences, derived directly from the clinical material (BE/21/2010 up) and after four cell culture passages (BE/21/2010 p4).

Nucleotide position	Genome region	BE/21/2010 up	BE/21/2010 p4	Length range inother HCMV strains
6,055–63	non-coding, UL	9–10 C’s	9–10 C’s	7–12
96,658–81	ncRNA4.9	23–24 T’s	23–24 T’s	7–24
99,184–207	UL69	5–8 CGG’s	8 CGG’s	2–8
231,849–60	non-coding, US	9–13 G’s	10–13 G’s	8–15
232,207–20	non-coding, US	11–15 G’s	11–15 G’s	9–15

(4) To characterize potential changes during further adaptation of HCMV to fibroblast replication, strains BE/9/2010 and BE/11/2010 were sampled and sequenced at different culture passages. Strain BE/9/2010 was sequenced after passage 2, 5, 7 and 11. Consensus sequences were derived independently. Consensus sequences for passages 2, 5 and 7 were identical, whereas passage 11 contained one substitution in gene UL44 (A128V), which encodes the DNA polymerase processivity subunit [46]. Analysis of the read alignments indicate that this mutation had arisen somewhere between passage 2 and 5 and was gradually becoming the dominant type at this position. At passage 2, all reads displayed the wild-type while at passage 5 the mutation was present in 3% of the reads. At passage 7 and 11, this fraction had risen to 33% and 77% respectively. This variability of UL44 has been shown before by Dargan *et al.*, albeit at different positions and at much higher passage numbers [34]. Subsequently, strain BE/11/2010 was sequenced after passage 2, 5 and 9. All derived consensus sequences were identical. To summarize, both strain’s sequences analyzed with the presented workflow were perfectly matching (up to passage 11), indicating that potential mutations during this period would not have a considerable impact on the overall consensus sequence.

It has been shown that gene RL13 and one of the genes of the UL128 locus (UL128, UL130 and UL131A; together UL128L) consistently mutate during passaging because of their inhibitory effect on HCMV replication in fibroblasts [34], [45]. Interestingly, none of the five strains that were sequenced in our study showed obvious gene-disrupting mutations in UL128L or RL13. This would indicate that the strains had not yet undergone these hallmark mutations that accompany the initial adaptation to growth in human fibroblasts and could therefore be considered genetically unaltered by cell culture. It cannot be excluded however, that some of these strains do contain mutations in UL128L or RL13. Mutations could be present at different positions in different members of the viral population, which would result in a wild-type consensus sequence, as was the case for RL13 in Merlin [45].

Taken together, these validation experiments indicate that the presented workflow had only a minimal impact on consensus sequences of the clinical isolates under study. Most of the differences detected between independent replicates could most likely be attributed to heterogeneity of repeat lengths in the original clinical isolates. The stability of sequences throughout these procedures shows that they are characteristic for the original strains present in clinical isolates.

### Genome Sequences Confirm Presence of Gene-disrupting Mutations in Clinical HCMV Isolates

Adaptation of HCMV strains to cell culture is accompanied by changes in the HCMV genome, including gene-disrupting mutations [6], [34]. More recently, evidence indicated that HCMV mutants could be present in unpassaged clinical isolates as well [17], [34]. Strain JP was sequenced without *in vitro* amplification and was mutated in genes RL5A and UL111A [17]. We analyzed strain BE/21/2010 directly from clinical material and identified disruptive mutations in RL5A, UL9 and UL150. Furthermore, we examined ORFs currently annotated on the HCMV reference strain Merlin for the presence of gene-disrupting mutations in the other four strains under study and found that genes RL5A, UL1, UL9 and UL111A could contain disruptive mutations (Table 3). Mutations in RL5A, UL1, UL9 and UL111A have been identified in earlier publications [17], [18], [34]. The transgenic strain CINCY+Towne (NCBI GenBank acc. no. GU980198) has a frameshift-inducing deletion in UL150, but this strain was passaged several times in human fibroblasts. To our knowledge this is the first report about a gene-disrupting mutation in UL150 present in an uncultured viral isolate. To rule out the possibility that mutations in strains BE/10/2010, BE/11/2010 and BE/27/2010 were acquired during passaging, viral genes of interest were PCR amplified and sequenced from the original clinical material (Table S6). All verified gene sequences from the clinical material corresponded to the sequences generated with NGS from the passaged material. Furthermore, identical mutations were present in distinct strains (Table 3). The fact that these mutations are conserved between independent and even geographically unrelated isolates provides a further indication of their widespread occurrence in clinical HCMV isolates.

**Table 3:** Gene-disrupting mutations in clinical HCMV strains.

Strain	RL5A	UL1	UL9	UL111A	UL150
BE/9/2010	wt	wt	wt	wt	wt
BE/10/2010	wt	wt	point mutation°	wt	wt
BE/11/2010	11 bp deletion°’	several point mutations*	wt	wt	wt
BE/21/2010	17 bp deletion”	wt	point mutation	wt	2 bp deletion
BE/27/2010	11 bp deletion°’	several point mutations°*	point mutation°	220 bp deletion°	wt
Other published full genome strains	JP, HAN13”	JHC	AF1	JP, PH	CINCY+Towne

wt = wild-type.

JP (GQ221975), HAN13 (GQ221973), JHC (HQ380895), AF1 (GU179291), PH (AC146904), CINCY+Towne (GU980198).

°Mutations verified by PCR amplification (Table S6) and Sanger sequencing of the viral gene in the original clinical material.

‘,”,*Identical mutations in unrelated strains.

HCMV gene family RL11 stands out in particular with several members (RL5A, RL6, UL1 and UL9) being suggested here and/or elsewhere to be mutated *in vivo* [17], [34], [47]. Most of these genes are hypervariable and their gene products are poorly characterized. UL1 encodes an envelope glycoprotein that was suggested to be a cell-type specific tropism factor [48], but for RL5A, RL6 and UL9 no functionality data are available. The same holds for gene UL150. Most interestingly, gene UL111A is mutated in strain BE/27/2010, the previously sequenced strains JP and PH and four isolates from renal transplant recipients [17], [49]. Strains BE/27/2010 and JP have deletions of 220 bp and 38 bp, which interfere with splicing of the second and first exon respectively. Strain PH has a substitution in the splice-acceptor site for the second exon. In the renal transplant recipients, three isolates (NCBI GenBank acc. no. EF488364-6) share a 5 bp deletion in the first exon, while a fourth isolate (EF535834) has a nonsense mutation in the first exon. UL111A encodes a viral interleukin-10 homolog, which has been shown to be involved in immune regulation, both during lytic and latent replication [50]. The observed existence of UL111A mutants in natural settings may have clinical significance, although more research is warranted to characterize the occurrence of mutations in different patient groups, both immunocompetent and immunocompromised. Interestingly, UL111A mutants have only been described in transplant recipients (BE/27/2010, PH and renal transplant isolates) or AIDS patients (JP), suggesting that the presence of these UL111A mutants could be associated with a defective immune system.

Our data indicate that the HCMV coding capacity is not fixed but can vary between different isolates. Additional full genome sequences from diverse patient groups and geographical areas are needed to characterize in further detail what ORFs can be mutated in clinical isolates, at what frequencies and in what patient groups.

## Conclusion

The introduction of a new generation of sequencing technologies with high-throughput capacities has immensely impacted the field of genomics. Previous publications have provided a snapshot of the possible applications in the field of HCMV genomics and transcriptomics [12], [13], [16]–[18], [33], [38]. We believe that the amplification, sequencing and analysis workflow that we present in this study can help to maximize the efficiency of sequencing HCMV strains in high-throughput. Given the large genetic background of HCMV, it could be interesting to routinely elucidate the complete sequence of strains that are used in mutational studies. This should no longer be considered as extremely laborious or costly. Additionally, the analysis of clinical HCMV isolates could assist in the refinement of the HCMV genetic map. It will provide a better knowledge of viral mutants and in which patient populations they are circulating. Finally, it could prove to be of value in the ongoing quest for genetic determinants of viral pathogenicity that has eluded scientists for more than a decade [37], [51], [52].

## Supporting Information

Table S1 Primers and probes for HCMV UL86 and human β-globin qPCR.
(DOCX)

Table S2 Performance of HCMV *de novo* genome assembly with 454 GS FLX and/or IGA datasets and different assembly software suites.
(XLSX)

Table S3 Primers and annealing temperatures for PCRs finishing the full genome sequences of strains BE/9/2010, BE/11/2010 and BE/21/2010.
(DOCX)

Table S4 *De novo* assembly of 454 GS FLX and IGA reads not mapping to the HCMV consensus sequence.
(DOCX)

Table S5 Consensus sequences of strains BE/10/2010 i1 – BE/10/2010 i2 and strains BE/27/2010 i1 – BE/27/2010 i2, derived from the same patient, only differed in homopolymer lengths.
(DOCX)

Table S6 Primers and annealing temperatures for PCRs amplifying mutated HCMV genes.
(DOCX)

## References

[1] Cannon MJ, Schmid DS, Hyde TB (2010) Review of cytomegalovirus seroprevalence and demographic characteristics associated with infection. Rev Med Virol 20: 202–213. 20564615 10.1002/rmv.655

[2] Britt W (2008) Manifestations of human cytomegalovirus infection: proposed mechanisms of acute and chronic disease. Curr Top Microbiol Immunol 325: 417–470. 18637519 10.1007/978-3-540-77349-8_23

[3] Murphy E, Shenk T (2008) Human cytomegalovirus genome. Curr Top Microbiol Immunol 325: 1–19. 18637497 10.1007/978-3-540-77349-8_1

[4] Chee MS, Bankier AT, Beck S, Bohni R, Brown CM, et al (1990) Analysis of the protein-coding content of the sequence of human cytomegalovirus strain AD169. Curr Top Microbiol Immunol 154: 125–169. 2161319 10.1007/978-3-642-74980-3_6

[5] Cha TA, Tom E, Kemble GW, Duke GM, Mocarski ES, et al (1996) Human cytomegalovirus clinical isolates carry at least 19 genes not found in laboratory strains. J Virol 70: 78–83. 8523595 10.1128/jvi.70.1.78-83.1996 PMC189790

[6] Prichard MN, Penfold ME, Duke GM, Spaete RR, Kemble GW (2001) A review of genetic differences between limited and extensively passaged human cytomegalovirus strains. Rev Med Virol 11: 191–200. 11376481 10.1002/rmv.315

[7] Davison AJ, Dolan A, Akter P, Addison C, Dargan DJ, et al (2003) The human cytomegalovirus genome revisited: comparison with the chimpanzee cytomegalovirus genome. J Gen Virol 84: 17–28. 12533697 10.1099/vir.0.18606-0

[8] Murphy E, Rigoutsos I, Shibuya T, Shenk TE (2003) Reevaluation of human cytomegalovirus coding potential. Proc Natl Acad Sci U S A 100: 13585–13590. 14593199 10.1073/pnas.1735466100 PMC263857

[9] Murphy E, Yu D, Grimwood J, Schmutz J, Dickson M, et al (2003) Coding potential of laboratory and clinical strains of human cytomegalovirus. Proc Natl Acad Sci U S A 100: 14976–14981. 14657367 10.1073/pnas.2136652100 PMC299866

[10] Dolan A, Cunningham C, Hector RD, Hassan-Walker AF, Lee L, et al (2004) Genetic content of wild-type human cytomegalovirus. J Gen Virol 85: 1301–1312. 15105547 10.1099/vir.0.79888-0

[11] Zhang G, Raghavan B, Kotur M, Cheatham J, Sedmak D, et al (2007) Antisense transcription in the human cytomegalovirus transcriptome. J Virol 81: 11267–11281. 17686857 10.1128/JVI.00007-07 PMC2045512

[12] Gatherer D, Seirafian S, Cunningham C, Holton M, Dargan DJ, et al (2011) High-resolution human cytomegalovirus transcriptome. Proc Natl Acad Sci U S A 108: 19755–19760. 22109557 10.1073/pnas.1115861108 PMC3241806

[13] Stern-Ginossar N, Weisburd B, Michalski A, Le VT, Hein MY, et al (2012) Decoding human cytomegalovirus. Science 338: 1088–1093. 23180859 10.1126/science.1227919 PMC3817102

[14] Mardis ER (2008) Next-generation DNA sequencing methods. Annu Rev Genom Hum G 9: 387–402. 10.1146/annurev.genom.9.081307.164359 18576944

[15] Bankier AT, Beck S, Bohni R, Brown CM, Cerny R, et al (1991) The DNA sequence of the human cytomegalovirus genome. DNA Seq 2: 1–12. 1666311 10.3109/10425179109008433

[16] Bradley AJ, Lurain NS, Ghazal P, Trivedi U, Cunningham C, et al (2009) High-throughput sequence analysis of variants of human cytomegalovirus strains Towne and AD169. J Gen Virol 90: 2375–2380. 19553388 10.1099/vir.0.013250-0 PMC2885757

[17] Cunningham C, Gatherer D, Hilfrich B, Baluchova K, Dargan DJ, et al (2010) Sequences of complete human cytomegalovirus genomes from infected cell cultures and clinical specimens. J Gen Virol 91: 605–615. 19906940 10.1099/vir.0.015891-0 PMC2885759

[18] Jung GS, Kim YY, Kim JI, Ji GY, Jeon JS, et al (2011) Full genome sequencing and analysis of human cytomegalovirus strain JHC isolated from a Korean patient. Virus Res 156: 113–120. 21255625 10.1016/j.virusres.2011.01.005

[19] Billiau A, Edy VG, Heremans H, Vandamme J, Desmyter J, et al (1977) Human Interferon - Mass-Production in a Newly Established Cell Line, Mg-63. Antimicrob Agents Ch 12: 11–15. 10.1128/aac.12.1.11 PMC352146 883813

[20] Sinzger C, Knapp J, Schmidt K, Kahl M, Jahn G (1999) A simple and rapid method for preparation of viral DNA from cell associated cytomegalovirus. J Virol Methods 81: 115–122. 10488769 10.1016/s0166-0934(99)00058-0

[21] Fronhoffs S, Totzke G, Stier S, Wernert N, Rothe M, et al (2002) A method for the rapid construction of cRNA standard curves in quantitative real-time reverse transcription polymerase chain reaction. Mol Cell Probes 16: 99–110. 12030760 10.1006/mcpr.2002.0405

[22] Chevreux B, Pfisterer T, Drescher B, Driesel AJ, Muller WE, et al (2004) Using the miraEST assembler for reliable and automated mRNA transcript assembly and SNP detection in sequenced ESTs. Genome Res 14: 1147–1159. 15140833 10.1101/gr.1917404 PMC419793

[23] Chevreux B, Wetter T, Suhai S (1999) Genome sequence assembly using trace signals and additional sequence information. Computer science and biology: proceedings of the German conference on bioinformatics (GCB) 99: 45–56.

[24] Zerbino DR, Birney E (2008) Velvet: algorithms for de novo short read assembly using de Bruijn graphs. Genome Res 18: 821–829. 18349386 10.1101/gr.074492.107 PMC2336801

[25] Machado M, Magalhaes WC, Sene A, Araujo B, Faria-Campos AC, et al (2011) Phred-Phrap package to analyses tools: a pipeline to facilitate population genetics re-sequencing studies. Investig Genet 2: 3. 10.1186/2041-2223-2-3 PMC3041995 21284835

[26] Kurtz S, Phillippy A, Delcher AL, Smoot M, Shumway M, et al (2004) Versatile and open software for comparing large genomes. Genome Biol 5: R12. 14759262 10.1186/gb-2004-5-2-r12 PMC395750

[27] Milne I, Bayer M, Cardle L, Shaw P, Stephen G, et al (2010) Tablet–next generation sequence assembly visualization. Bioinformatics 26: 401–402. 19965881 10.1093/bioinformatics/btp666 PMC2815658

[28] Milne I, Stephen G, Bayer M, Cock PJ, Pritchard L, et al.. (2012) Using Tablet for visual exploration of second-generation sequencing data. Brief Bioinform. 10.1093/bib/bbs012 22445902

[29] Katoh K, Toh H (2010) Parallelization of the MAFFT multiple sequence alignment program. Bioinformatics 26: 1899–1900. 20427515 10.1093/bioinformatics/btq224 PMC2905546

[30] Tamura K, Peterson D, Peterson N, Stecher G, Nei M, et al (2011) MEGA5: molecular evolutionary genetics analysis using maximum likelihood, evolutionary distance, and maximum parsimony methods. Mol Biol Evol 28: 2731–2739. 21546353 10.1093/molbev/msr121 PMC3203626

[31] Camacho C, Coulouris G, Avagyan V, Ma N, Papadopoulos J, et al (2009) BLAST+: architecture and applications. BMC Bioinformatics 10: 421. 20003500 10.1186/1471-2105-10-421 PMC2803857

[32] Huson DH, Mitra S, Ruscheweyh HJ, Weber N, Schuster SC (2011) Integrative analysis of environmental sequences using MEGAN4. Genome Res 21: 1552–1560. 21690186 10.1101/gr.120618.111 PMC3166839

[33] Renzette N, Bhattacharjee B, Jensen JD, Gibson L, Kowalik TF (2011) Extensive genome-wide variability of human cytomegalovirus in congenitally infected infants. PLoS Pathog 7: e1001344. 21625576 10.1371/journal.ppat.1001344 PMC3098220

[34] Dargan DJ, Douglas E, Cunningham C, Jamieson F, Stanton RJ, et al (2010) Sequential mutations associated with adaptation of human cytomegalovirus to growth in cell culture. J Gen Virol 91: 1535–1546. 20479471 10.1099/vir.0.018994-0 PMC3052722

[35] Lasken RS (2009) Genomic DNA amplification by the multiple displacement amplification (MDA) method. Biochem Soc Trans 37: 450–453. 19290880 10.1042/BST0370450

[36] Direito SOL, Zaura E, Little M, Ehrenfreund P, Röling WFM (2014) Systematic evaluation of bias in microbial community profiles induced by whole genome amplification. Environ Microbiol 16(3): 643–657. 24372985 10.1111/1462-2920.12365

[37] Puchhammer-Stockl E, Gorzer I (2011) Human cytomegalovirus: an enormous variety of strains and their possible clinical significance in the human host. Future Virol 6: 259–271.

[38] Gorzer I, Guelly C, Trajanoski S, Puchhammer-Stockl E (2010) Deep sequencing reveals highly complex dynamics of human cytomegalovirus genotypes in transplant patients over time. J Virol 84: 7195–7203. 20463084 10.1128/JVI.00475-10 PMC2898262

[39] Li Z, Chen Y, Mu D, Yuan J, Shi Y, et al (2012) Comparison of the two major classes of assembly algorithms: overlap-layout-consensus and de-bruijn-graph. Brief Funct Genomics 11: 25–37. 22184334 10.1093/bfgp/elr035

[40] Archer J, Baillie G, Watson SJ, Kellam P, Rambaut A, et al (2012) Analysis of high-depth sequence data for studying viral diversity: a comparison of next generation sequencing platforms using Segminator II. BMC Bioinformatics 13: 47. 22443413 10.1186/1471-2105-13-47 PMC3359224

[41] Peng Y, Leung HC, Yiu SM, Chin FY (2012) IDBA-UD: a de novo assembler for single-cell and metagenomic sequencing data with highly uneven depth. Bioinformatics 28: 1420–1428. 22495754 10.1093/bioinformatics/bts174

[42] Bankevich A, Nurk S, Antipov D, Gurevich AA, Dvorkin M, et al (2012) SPAdes: a new genome assembly algorithm and its applications to single-cell sequencing. J Comput Biol 19: 455–477. 22506599 10.1089/cmb.2012.0021 PMC3342519

[43] Magoc T, Pabinger S, Canzar S, Liu X, Su Q, et al (2013) GAGE-B: an evaluation of genome assemblers for bacterial organisms. Bioinformatics 29: 1718–1725. 23665771 10.1093/bioinformatics/btt273 PMC3702249

[44] Nurk S, Bankevich A, Antipov D, Gurevich AA, Korobeynikov A, et al (2013) Assembling single-cell genomes and mini-metagenomes from chimeric MDA products. J Comput Biol 20: 714–737. 24093227 10.1089/cmb.2013.0084 PMC3791033

[45] Stanton RJ, Baluchova K, Dargan DJ, Cunningham C, Sheehy O, et al (2010) Reconstruction of the complete human cytomegalovirus genome in a BAC reveals RL13 to be a potent inhibitor of replication. J Clin Invest 120: 3191–3208. 20679731 10.1172/JCI42955 PMC2929729

[46] Weiland KL, Oien NL, Homa F, Wathen MW (1994) Functional analysis of human cytomegalovirus polymerase accessory protein. Virus Res 34: 191–206. 7856311 10.1016/0168-1702(94)90124-4

[47] Sekulin K, Gorzer I, Heiss-Czedik D, Puchhammer-Stockl E (2007) Analysis of the variability of CMV strains in the RL11D domain of the RL11 multigene family. Virus Genes 35: 577–583. 17823862 10.1007/s11262-007-0158-0

[48] Shikhagaie M, Merce-Maldonado E, Isern E, Muntasell A, Alba MM, et al (2012) The human cytomegalovirus-specific UL1 gene encodes a late-phase glycoprotein incorporated in the virion envelope. J Virol 86: 4091–4101. 22345456 10.1128/JVI.06291-11 PMC3318608

[49] Garrigue I, Corte MF, Magnin N, Couzi L, Capdepont S, et al (2007) Variability of UL18, UL40, UL111a and US3 immunomodulatory genes among human cytomegalovirus clinical isolates from renal transplant recipients. J Clin Virol 40: 120–128. 17827058 10.1016/j.jcv.2007.06.015

[50] Slobedman B, Barry PA, Spencer JV, Avdic S, Abendroth A (2009) Virus-encoded homologs of cellular interleukin-10 and their control of host immune function. J Virol 83: 9618–9629. 19640997 10.1128/JVI.01098-09 PMC2747999

[51] Pignatelli S, Dal Monte P, Rossini G, Landini MP (2004) Genetic polymorphisms among human cytomegalovirus (HCMV) wild-type strains. Rev Med Virol 14: 383–410. 15386592 10.1002/rmv.438

[52] Puchhammer-Stockl E, Gorzer I (2006) Cytomegalovirus and Epstein-Barr virus subtypes–the search for clinical significance. J Clin Virol 36: 239–248. 16697698 10.1016/j.jcv.2006.03.004

