# Supplementary material for: A Method Enabling High-Throughput Sequencing of Human Cytomegalovirus Complete Genomes from Clinical Isolates
Source: PLoS One. 2014 Apr 22;9(4):e95501. doi: 10.1371/journal.pone.0095501 (PMC3995935; doi:10.1371/journal.pone.0095501)
Supplement: Table S1 — Primers and probes for HCMV UL86 and human β-globin qPCR. (DOCX) [file pone.0095501.s001.docx]

Table S1. Primers and probes for HCMV UL86 and human β-globin qPCR.

|  | HCMV UL86 qPCR | Human β-globin qPCR |
| --- | --- | --- |
| Forward primer | CAC GGT CCC GGT TTA GCA | ACA CAA CTG TGT TCA CTA GC |
| Reverse primer | CGT AAC GTG GAC CTG ACG TTT | TGG TCT CCT TAA ACC TGT CTT G |
| Probe | FAM - TGT AAC CGC GAT CCT CGG GCA GAT A - TAMRA | JOE - CCA CCA ACT TCA TCC ACG TTC ACC TT - TAMRA |
