# Supplementary material for: A Method Enabling High-Throughput Sequencing of Human Cytomegalovirus Complete Genomes from Clinical Isolates
Source: PLoS One. 2014 Apr 22;9(4):e95501. doi: 10.1371/journal.pone.0095501 (PMC3995935; doi:10.1371/journal.pone.0095501)
Supplement: Table S3 — Primers and annealing temperatures for PCRs finishing the full genome sequences of strains BE/9/2010, BE/11/2010 and BE/21/2010. (DOCX) [file pone.0095501.s003.docx]

Table S3. Primers and annealing temperatures for PCRs finishing the full genome sequences of strains BE/9/2010, BE/11/2010 and BE/21/2010.

| Strain |  | Forward primer | Reverse primer | Annealing Temperature |
| --- | --- | --- | --- | --- |
| BE/9/2010 | 1 | TCT GCG TGT GTC TTC GAC G | TGG CTT TTA TAG GCA GCG ACG | 52°C |
| BE/9/2010 | 2 | TGT CTC CGT CCC CAC CAC C | GGG CAC ACT GCT TCC ATC C | 55.5°C |
| BE/11/2010 | 1 | GGA CTC CAC AAA CCA CAC | GTG TTC TTC GTT GGC GTG | 50°C |
| BE/11/2010 | 2 | CTG TTC TGA TAG GGG CAC C | TTT TAT AGG CAG CGG CGT G | 50°C |
| BE/11/2010 | 3 | CAA CAC GCC GCT AAT GCG | ACG AAC AGC AAC TCC CAG G | 52°C |
| BE/21/2010 | 1 | ACG TGT TTG GAA CTC TGT CC | TAC AAC AGG GAA GGA TAC GG | 53.5°C |
| BE/21/2010 | 2 | TAT CAT CGC TGT AAC ACA GC | TGA GCT TAA CTT GAT GAC GC | 51°C |
| BE/21/2010 | 3 | ATC TGG CTG AGC TGT ACG TC | TTA AAG GCA AAT GCA CCT CGT C | 55°C |
| BE/21/2010 | 4 | TTT GTC GTC CCC GCT GCA G | CGT GTT GTG TCC GAC GCT G | 56°C |
