# Supplementary material for: A Method Enabling High-Throughput Sequencing of Human Cytomegalovirus Complete Genomes from Clinical Isolates
Source: PLoS One. 2014 Apr 22;9(4):e95501. doi: 10.1371/journal.pone.0095501 (PMC3995935; doi:10.1371/journal.pone.0095501)
Supplement: Table S4 — De novo assembly of 454 GS FLX and IGA reads not mapping to the HCMV consensus sequence. (DOCX) [file pone.0095501.s004.docx]

Table S4. *De novo* assembly of 454 GS FLX and IGA reads not mapping to the HCMV consensus sequence°.

| Strain | Isolate and/or passage number | # *de novo* contigs | # contigs with no hits or low complexity hits | # contigs of human origin | # contigs of HCMV origin | # contigs of other origins |
| --- | --- | --- | --- | --- | --- | --- |
| Merlin |  | 29 | 22 | 7 | 0 | 0 |
| BE/9/2010 | p2 | 111 | 59 | 47 | 0 | 5 (*Pseudomonas aeruginosa*) |
| BE/9/201 | p5 | 28 | 12 | 16 | 0 | 0 |
| BE/9/2010 | p7 | 102 | 50 | 52 | 0 | 0 |
| BE/9/2010 | p11 | 655 | 359 | 291 | 0 | 5 (*Rhizobiales*) |
| BE/10/2010 | i1 p2 | 10 | 10 | 0 | 0 | 0 |
| BE/10/2010 | i2 p2 | 10 | 10 | 0 | 0 | 0 |
| BE/11/2010 | p2 | 41 | 23 | 18 | 0 | 0 |
| BE/11/2010 | p5 | 114 | 71 | 43 | 0 | 0 |
| BE/11/2010 | p9 | 220 | 130 | 90 | 0 | 0 |
| BE/21/2010 | up | 5002 | 4013 | 566 | 0 | 14 (*Papillomaviridae*),  36 (*Proteobacteria*),  90 (*Firmicutes*),  53 (*Bacteroidales*),  230 (*Actinomycetales*) |
| BE/21/2010 | p4 | 176 | 116 | 25 | 35 | 0 |
| BE/27/2010 | i1 p4 | 627 | 378 | 239 | 10 | 0 |
| BE/27/2010 | i2 p4 | 109 | 72 | 0 | 37 | 0 |

i = isolate number

p = passage number

up = unpassaged

° Contig origins were analyzed using the *BLAST+* application and *MEGAN4*.
