# Supplementary material for: A Method Enabling High-Throughput Sequencing of Human Cytomegalovirus Complete Genomes from Clinical Isolates
Source: PLoS One. 2014 Apr 22;9(4):e95501. doi: 10.1371/journal.pone.0095501 (PMC3995935; doi:10.1371/journal.pone.0095501)
Supplement: Table S5 — Consensus sequences of strains BE/10/2010 i1 – BE/10/2010 i2 and strains BE/27/2010 i1 – BE/27/2010 i2, derived from the same patient, only differed in homopolymer lengths. (DOCX) [file pone.0095501.s005.docx]

Table S5. Consensus sequences of strains BE/10/2010 i1 – BE/10/2010 i2 and strains BE/27/2010 i1 – BE/27/2010 i2, derived from the same patient, only differed in homopolymer lengths.

| Strain | Nucleotide position | Genome region | Number and type of nucleotides | Length range in other HCMV strains |
| --- | --- | --- | --- | --- |
| BE/10/2010 | 93,542-51 | oriLyt | 9-10 C’s | 9-11 |
| BE/10/2010 | 96,841-59 | ncRNA4.9 | 16-19 T’s | 7-24 |
| BE/10/2010 | 231,169-80 | non-coding, US | 12-14 G’s | 10-15 |
| BE/27/2010 | 230,830-41 | non-coding, US | 11-12 G’s | 10-15 |
