# Supplementary material for: A Method Enabling High-Throughput Sequencing of Human Cytomegalovirus Complete Genomes from Clinical Isolates
Source: PLoS One. 2014 Apr 22;9(4):e95501. doi: 10.1371/journal.pone.0095501 (PMC3995935; doi:10.1371/journal.pone.0095501)
Supplement: Table S6 — Primers and annealing temperatures for PCRs amplifying mutated HCMV genes. (DOCX) [file pone.0095501.s006.docx]

Table S6. Primers and annealing temperatures for PCRs amplifying mutated HCMV genes.

| Gene | Forward primer | Reverse primer | Annealing Temperature |
| --- | --- | --- | --- |
| RL5A | AAC GTA TGA TAT TTG TTC CC | GAA CCG GTC CAG TCA CA | 51°C |
| UL1 | TRA YAT GCA CAT CAA TAA AC | AAY GAR GGA AAT TCC ACT C | 49°C |
| UL9 | ATY TGT CTR CRA GCA CCT C | TGA CAG TGA CCT CCA TAC | 49°C |
| UL111A | CAT CAT AAC ATA AAG GAC CAC C | CAA CAC CCA CAA ACA ACG TC | 53°C |
